# A Bioinformatics Systems Biology Analysis of the Current Oral Proteomic Biomarkers and Implications for Diagnosis and Treatment of External Root Resorption

**DOI:** 10.3390/ijms22063181

**Published:** 2021-03-20

**Authors:** Mahmoud Mona, Zunnaira Abbasi, Firas Kobeissy, Abdulrahman Chahbandar, Roberta Pileggi

**Affiliations:** 1 Department of Endodontics, University of Florida College of Dentistry, Gainesville, FL 32610, USA; ZAbbasi@dental.ufl.edu (Z.A.); AChahbandar@dental.ufl.edu (A.C.); RPILEGGI@dental.ufl.edu (R.P.); 2 Department of Emergency Medicine, McKnight Brain Institute, University of Florida, Gainesville, FL 32610, USA; firasko@ufl.edu

**Keywords:** external root resorption, invasive cervical resorption, dentin sialoprotein, dentin phosphoproteins, dentin sialophosphoprotein, dentin matrix acidic phosphoprotein 1, dental trauma and biomarkers

## Abstract

External root resorption (ERR) is a silent destructive phenomenon detrimental to dental health. ERR may have multiple etiologies such as infection, inflammation, traumatic injuries, pressure, mechanical stimulations, neoplastic conditions, systemic disorders, or idiopathic causes. Often, if undiagnosed and untreated, ERR can lead to the loss of the tooth or multiple teeth. Traditionally, clinicians have relied on radiographs and cone beam computed tomography (CBCT) images for the diagnosis of ERR; however, these techniques are not often precise or definitive and may require exposure of patients to more ionizing radiation than necessary. To overcome these shortcomings, there is an immense need to develop non-invasive approaches such as biomarker screening methods for rapid and precise diagnosis for ERR. In this review, we performed a literature survey for potential salivary or gingival crevicular fluid (GCF) proteomic biomarkers associated with ERR and analyzed the potential pathways leading to ERR. To the best of our knowledge, this is the first proteomics biomarker survey that connects ERR to body biofluids which represents a novel approach to diagnose and even monitor treatment progress for ERR.

## 1. Introduction

Inflammatory root resorption can occur inside the dental pulp, known as internal resorption, or on the root outer surface, called ERR (external root resorption). ERR is a type of root resorption that involves periodontal ligament damage in addition to dentin and cementum destruction. ERR can occur due to physiologic or pathological processes such as trauma, orthodontic tooth movement, bleaching, a pathology, or it can also be idiopathic in nature. For root resorption to occur, there has to be loss of the protective layer of precementum and predentin along with inflammation at the unprotected root surface [1]. ERR potentially causes extensive tooth destruction leading to tooth loss. The common types of ERR involve root destruction due to trauma or rapid orthodontic movement associated with bacterial invasion of the dentinal tubules triggering an inflammatory process [2,3]. Invasive cervical resorption (ICR), on the other hand, is considered a variant of ERR characterized by its subepithelial location, invasive nature, and sometimes a pink discoloration of the clinical crown [4]. While the process of ICR begins just below the gingival epithelial attachment on the tooth, the other type of ERR can occur on any area of the root surface [5]. Both types of ERR are usually painless to the patient and are commonly discovered as an incidental finding. Within the defect, dentin is replaced with fibrovascular tissue derived from the periodontal ligament and multinucleated giant cells are also seen [6]. Early detection and proper management of this condition would provide a better prognosis for tooth restoration and reduce the associated oral dysfunction due to this aggressive disease. 

One of the novel diagnostic methods is the application of biomarker assessment for disease detection. A biological marker or biomarker is a biological substance that can be measured quantitatively and plays an important role in diagnosing the onset of certain diseases and tracks their progression and treatment. It also helps to predict patient susceptibility to a certain type of disease. From a diagnostic standpoint, the use of biomarkers for the early detection of oral diseases such as root resorptions among others has generated a huge interest in recent years and has been increasingly investigated in the dental field. Biomarkers have been found to be present in different bodily fluids such as saliva, GCF, blood, or urine [7], but for this review we will focus on biomarkers detected in the GCF and saliva. There is currently no specific salivary or GCF diagnostic marker that allows for early diagnosis of this disease, an area of study that should be explored and researched more for timely and early critical diagnosis of root resorptions. 

In this review, we searched PubMed for experimental and clinical studies published up to January of 2021 in English. An example of search free terms used was “root resorption” and “biomarker”, which retrieved approximately 108 articles. Other search words used include “inflammatory root resorption”, “idiopathic root resorption”, and “cervical root resorption”. We then read and filtered the eligible papers based on their methodology, results, and conclusions. Consequently, the identified biomarkers were further analyzed for their possible protein–protein interaction with common inflammatory and non-inflammatory biomarkers using a high throughput proteomic platform, Pathway Studio^®^ (version 11.0; Ariadne Genomics/Elsevier Inc., Rockville, MD, USA) [8,9].

## 2. Etiology and Pathogenesis 

The process of root resorption is associated with the loss of cemental layer integrity below the epithelial attachment. This layer is known to function in preventing osteoclasts from resorbing and remodeling the underlying dentin [5]. Destruction and deterioration of this thin layer of cementum along with bacterial invasion is what leads to ERR. In most cases, ERR is asymptomatic and detected by incidental findings. The majority of external root resorption studies have associated it with traumatic tooth injury or orthodontic tooth movement [10,11]. Trauma or stress associated with orthodontic forces cause injury to the cementum layer leading to bacterial infection and inflammatory reaction [12]. As a result, osteoclasts are activated leading to dentinal resorption and later to bone replacement. In that case, it becomes external replacement resorption replacing the dental root with bony tissues [13].

On the other hand, ICR occurs in the cervical third of the root with no known direct cause. However, several studies have proved its association with systemic conditions such as Paget’s disease, Stevens-Johnson syndrome, Papillon–Lefevre syndrome, osteogenesis imperfecta, hypophosphatemia, hepatic disease, hormonal disturbance, hyperparathyroidism, bone dysplasia, hemifacial atrophy, and renal disease [14]. One known contributing factor to ICR is intracoronal bleaching. The bleaching agents used in these procedures, such as 30% hydrogen peroxide, can irritate the periodontal ligament and interrupt the root surface structure by penetrating through the dentin from the inside of the pulp chamber [15]. 

Furthermore, some studies have indicated that a familial pattern is noted in a special cohort of patients warranting further investigation on the possible genetic nature of ICR [16,17]. In some cases, there is a noted predilection toward a younger female population [18]. Elevated serum levels of alkaline phosphatase associated with Paget’s disease have been also observed in cases of ERR [19]. Pregnancy has also been postulated to be another contributing factor in the initiation or the occurrence of ERR [20]. In another case, when no other contributors were identified, chemotherapy was thought to be related to multiple ICRs in a patient years after treatment [21]. In another case study, it was shown that there may be a possible association between hepatitis B viral infection and increased osteoclastic activity [22]. Other external forces such as playing wind instruments have been associated with ERR. One study has shown a correlation in three clinical cases of wind instrument players with ICR [23]. Herpes zoster, on the other hand, has been suggested as a potential cause for ERR [24]. Similarly, other severe viral diseases occurring earlier in a patient’s life, such as whooping cough and meningitis, have shown to be also associated with ERR [25]. A comparison study found that ERR has similar histological, clinical, and radiographical presentations as a disease found in many domestic and wild cats called feline odontoclastic resorptive lesions suggestive of a possible viral contraction from cats [26]. Such multifactorial disease imposes a great diagnostic burden on clinicians. Thus, a definitive and unique diagnostic biomarker would provide a significant tool for early disease detection and more definitive diagnosis.

## 3. Diagnosis

ERR occurrence is usually not associated with pain or symptoms. It is often discovered incidentally or when the lesions have become extensive to stimulate nearby nerve fibers. Incidental findings from routine radiographs include a radiolucency originating from the cementoenamel junction that is either well-defined or molted in appearance [27]. In terms of clinical presentation, when the resorption is cervical and extensive, it can appear as a pink hue area in the cervical region or cavitation; this pink hue is due to the blood and fibrous tissue, filling the cavity created in cases of ICR [27,28]. In most cases, the lesion will extend to include the whole cervical region of the tooth [18]. Some clinical cases report normal associated periodontal or endodontic findings such as no mobility (unless lesions are extensive), probing depths of 3 mm or less, no bleeding upon probing, pulp testing within normal limits using refrigerant, and normal percussion test [29]. A pattern that is seen in some cases is the ingrowth of bone or tissue into the lesions [28,29]. Resorption in the lateral dental root may be associated with deep periodontal probing [30]. A CBCT image may be utilized as a diagnostic tool to identify the lesion’s true size, location, and extension [29]. The CBCT technology allows for the identification of buccal and lingual lesions that may not be apparent on periapical or panoramic radiographs [29]. However, in another clinical case, conventional periapical radiographs were not able to detect lesions on the buccal or lingual surfaces that are 0.6mm or smaller in diameter, while lesions of 1.8mm diameter or bigger were detected 74%–78% of the times [28]. The two-dimensional nature of a panoramic X-ray limits its diagnostic abilities in comparison to the three-dimensional analysis and slice capabilities of CBCT. Patients experiencing ERR can be diagnosed when the legion is large enough to be detected on CBCT or extensive enough to cause symptoms [28].

Thus, early detection of this lesion is impossible without a dental-specific biological marker(s). Such markers may be present in the patient’s saliva or GCF. Novel biomarker assays are considered an unmet need in the field of dental disease to allow for early detection and management of this detrimental condition. Thus, in this review we will survey and analyze all potential salivary and GCF markers associated with ERR. Furthermore, a high throughput systems biology bioinformatics analysis will be performed to detect any underlying mechanisms involved and the possible interaction between the found specific markers and upregulated inflammatory markers. 

## 4. Biomarkers Associated with ERR

GCF and saliva are currently considered the most commonly used fluids for the detection of biomarkers in oral diseases, and this may have significant applications across the field of dentistry. It is composed of substances derived from serum, host inflammatory cells, structural cells of the periodontium, and oral bacteria. It can be isolated from the healthy sulcus in small amounts. In certain inflammatory conditions, multiple pathological biomarkers have been detected in the GCF [25]. Monitoring the presence of certain biomarkers in GCF can be of potential value in diagnosing dental diseases such as ERR that can highlight a successful treatment trajectory.

GCF biomarker detection has already been employed to clinically track osteoclast activity, bone remodeling, and ERR occurring in particular during orthodontic treatment [31]. George and Evans tested the hypothesis that during root resorption organic proteins and cytokines from the surrounding bone and dentin are released into the gingival crevice. Findings from their study confirmed the presence of dentin matrix acidic phosphoprotein 1 (DMP-1) as one of the unique markers [32]. In another study demonstrating the various degrees of resorption, 2D gel electrophoresis analysis detected the presence of dentin phosphoproteins (DPP) in the GCF of patients with ERR which were validated via Western blot assay [32]. 

In Table 1, we summarize the identified proteins along with the associated pathways with the research studies cited. Of the dentin-related proteins, dentin sialoproteins (DSP) and DPP are two domains of a larger protein, dentin sialophosphoprotein (DSPP) [33]. DPP and DSP are dentin-specific non-collagenous proteins found predominantly in dentin. They are secreted by the dental pulp in the dentinal matrix and are most abundant in the presence of root resorption as compared to different biomarkers commonly found during physiological bone resorption. Mah and Prasad were among the first to detect these biomarkers in the GCF of patients with root resorption, specifically orthodontically induced resorption. Through their study, they were able to demonstrate that detection of these biomarkers in the GCF may have several advantages, such as providing high sensitivity diagnosis, avoiding harmful radiation risks of X-rays, and applying non-invasive approaches for the testing and monitoring of resorption progression. Another key finding was that DPP levels were higher in the two resorption groups than in the third control group, thus confirming DPP as a potential biomarker for ERR [34]. However, a drawback of this study was that they detected DPP in physiological levels of this protein as well [35]. Of note, other studies have not differentiated the types of proteins seen in physiological vs. pathological root resorptions. Therefore, quantitative longitudinal studies are needed to determine the presence of various levels of these biomarkers in cases showing the different types of root resorption [36].

The role of DSP has been found to be specific to dentin, proposing it as a predictive biomarker to diagnose root resorption. Its levels have been found to be raised in teeth undergoing physiological root resorption as well as in teeth undergoing orthodontic treatment [44]. These results highlight the potential of measuring levels of DSP in GCF as a biomarker to monitor root resorption. However, in addition to dentin, DSP can also originate from bone and cementum [44]. Thus, its level of secretion may be associated with resorption activity levels, which may be utilized to distinguish the disease from healthy concentrations of DSP in GCF as investigated in later studies. 

Balducci et al. detected high levels of DSP and DPP in the GCF of patients undergoing root resorption and showed that their concentrations were significantly higher in the control group. They found a significant difference in concentration of DSP and DPP in the severe (>2 mm root shortening) vs. mild (<2 mm root shortening) groups demonstrating these markers to be suitable for monitoring root resorption prior to its appearance on radiographs. A surprising outcome from their study was the presence of DSP and DPP in the control group as a background. This may be caused by physiologic root resorption of control teeth that was undiagnosed initially in the control group. They also noted levels of another protein, DMP-1, to be higher in the test group as compared to the control group, suggesting DMP-1 to be another potential GCF biomarker for root resorption. Interestingly, DMP-1 was detected even in mild levels of ERR [48]. Lombardo et al. developed a promising method for more efficient DSP quantification using a micro-bead approach compared to the conventional ELISA which would help in detecting lower levels of DSP in GCF [45]. 

Less specific markers have also been found in association with ERR. These include the receptor activator of nuclear factor-kappa-Β ligand (RANKL) and Osteoprotegerin (OPG). The OPG/RANKL/RANK system contributes to root resorption during orthodontic tooth movement and after traumatic injury [13]. Authors have speculated that determining the levels of OPG and RANKL in GCF correlate to the degree of root resorption during orthodontic tooth movement. This has been demonstrated using animal subjects and highlights an inductive role of this system in ERR. However, these are the mediators for the physiological bone remodeling and are not specific for ERR [13,40]. 

Interestingly, some other inflammatory markers have been found in cases of ERR. Increased levels of inflammatory cytokines have been detected in the GCF during root resorption. Among them are the interleukin-1beta (IL-1B), interleukin-6 (IL-6), and tumor necrosis factor-alpha (TNF-α). IL-1B inflammatory cytokine is secreted by osteoclasts due to traumatic impact or forces of orthodontic appliances [41,49]. In addition, TNF-α in the presence of RANKL can influence osteoclast differentiation and induce bone resorption. This is one of the direct causes of inflammatory reaction leading to root resorption. An immediate release of IL-1B and TNF-α due to orthodontic forces reaches its peak within 24 h of orthodontic adjustment [12]. This inflammatory process is required for successful tooth movement during orthodontic treatment; however, sudden or excessive forces may result in ERR.

Recently, novel biomarkers such as microRNAs (miRNAs) have gained significant interest in clinical research in recent years. Its role as a biomarker in the diagnosis of certain cancers, autoimmune diseases, and inflammatory diseases has already been well-established. Interestingly, several microRNA molecules have been found to be present in the GCF during root resorption [39]. Altawasuwan et al. have analyzed secretory miRNA 29 and confirmed its presence in GCF by Western blot and real-time PCR [47]. Increased levels of miRNA-29B have been detected during osteoclast differentiation in TNF alpha/RANKL-treated cells. This indicates that miRNA has a significant role in TNF-α-mediated osteoclast differentiation and OPG inhibition [50]. miRNA 29 was also found to induce MMP9 secretion amplifying dentinal and cementum destruction [51].

**Figure 1:**
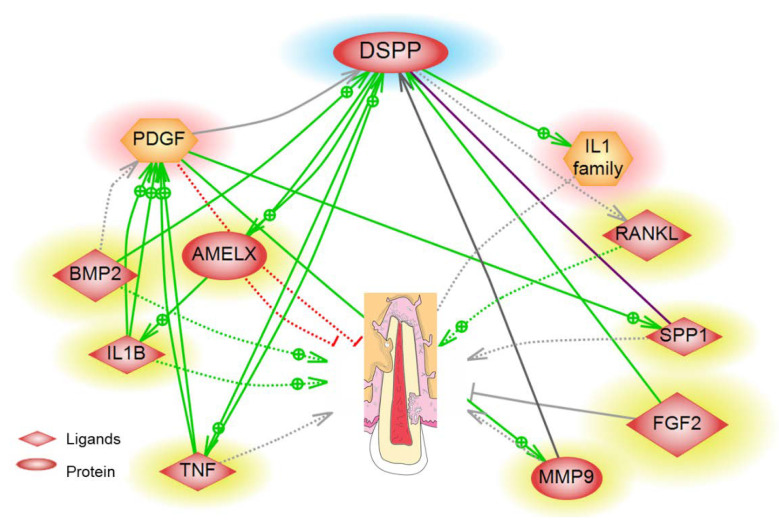
Systems biology analysis of the direct and indirect association between dentin-specific dentin sialophosphoprotein (DSPP) and ERR and any possible mediator proteins. A yellow halo represents a protein, and red represents a functional class.

Further bioinformatics analysis conducted by our team using the high throughput Pathway Studio Software on proteins involved in ERR have predicted the possible pathway of dentin specific, DSPP, through multiple inflammatory mediators leading to ERR. For example, as shown in Figure 1, DSPP induces secretion of the odontoblast regulatory factor RANKL leading to root resorption [38,52]. DSPP is cleaved to its two functional domains, DPP and DSP, by matrix metallopeptidase 9 (MMP9) as reported by Yuan et al. [53]. Furthermore, the DPP was found to enhance neutrophil migration by inducing the secretion of TNF-α and IL-1B [43,46]. High levels of bone morphogenetic protein 2 (BMP-2) also induce dental root resorption; however, a low level was found to aid in cementum regeneration, thus preventing resorption [54,55]. While the biomarkers we have discussed so far induced root resorption, our proteomic functional analysis and survey of the literature revealed some proteins that reduce resorptive activity such as platelet-derived growth factor (PDGF) and amelogenin X (AMELX) [56]. Noda et al. have shown that PDGF reduces the incidence of root resorption in the tooth re-implantation model in dogs [57], and Yagi et al. have proved that AMELX prohibits root resorption [56]. In addition, dental autotransplantation in dogs showed us the importance of FGF2 in inhibiting root resorption [58,59]. Multiple biomarkers for disease and health have been reported, but a quantitative assay is yet to be invented to allow for the detection of ERR activity in GCF. 

## 5. Treatment Options for ERR

**Figure 2:**
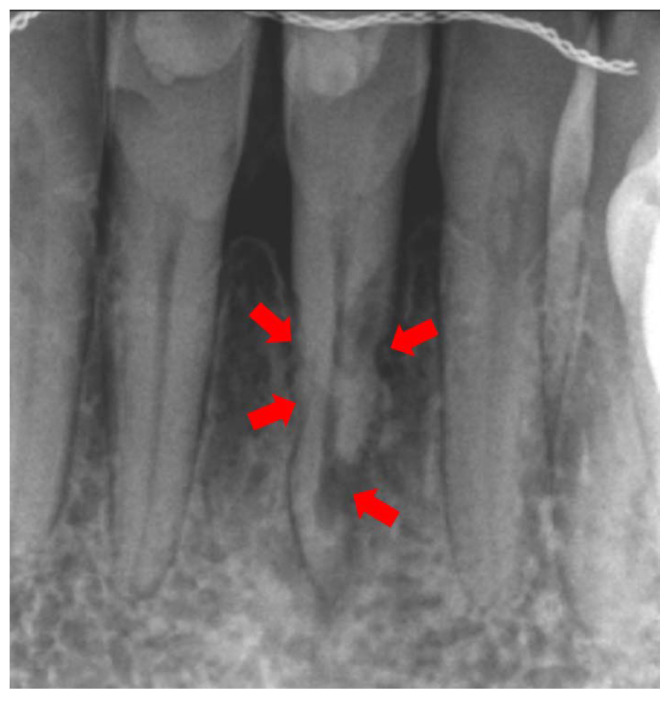
Periapical radiograph of tooth #24. Extensive resorptive defect observed on multiple areas of the dental root. The prognosis for this tooth was deemed guarded due to advanced ERR.

Based on the above literature, early detection of ERR is critical in controlling the spread of the disease in the dental root thus avoiding dental morbidity. The treatment goal in ERR is to excavate the resorptive defect, stop the progress of these lesions, and restore the defect with filling material. A delay in timely treatment can result in resorption perforating through the root canal wall, at which point endodontic treatment is indicated in addition to the root restoration. The usual approach for root restoration is performed by removing granulation tissue and treating the cavity with 90% trichloric acid (TSA) which promotes coagulation necrosis of the tissue without damaging the periodontal structures. Then, the cavity may be restored with aesthetic composite resin or glass ionomer cement. Biodentin and Mineral Trioxide Aggregate (MTA) have been shown to be promising materials for the restoration of such defects [60]. Sharma et al. and Wu J et al., in their respective studies, proved the biocompatibility and long-term stability in ERR cases treated with MTA [61,62]. Any delay in restoring the resorptive defect can deem the tooth non-restorable and require eventual extraction. For example, Figure 2 is a periapical radiograph of tooth #24 in a patient who presented to the Endodontics Department at the University of Florida College of Dentistry with ERR. In this image, extensive resorptive lesions reaching the dental pulp on #24 were caused by a traumatic injury 3 years prior to diagnosis. The status of the tooth is detrimental due to the large multiple lesions compromising the root structure and the root canal system. Had the patient been screened for ERR biomarkers during her routine dental exams, ERR could have been detected earlier with a much better prognosis. 

## 6. Conclusions

Taken together, this comprehensive review coupled with the data analysis demonstrate that the early detection of ERR is critical in the prevention of teeth loss. A novel non-invasive method for early disease detection is crucial for treatment success. Dentin-specific markers such as DMP, DSPP, and its functional domains DPP and DSP, in addition to inflammatory markers such as TNF- α, IL-6 and IL1-B, and MicroRNA- 29, are promising GCF biomarkers for ERR. In the future, such markers may be used in developing an exam kit for the detection of resorption biomarkers in GCF in a non-invasive manner. The field of proteomic biomarkers is still in its infancy in the area of dental disorders; such a diagnostic approach has excellent potential as it can prevent unnecessary invasive procedures, such as X-ray radiographs. It has a promising future use in patients’ routine dental care and can eventually have a significant impact on the overall health care system. 

## Figures and Tables

**Table 1:** Multiple GCF (gingival crevicular fluid) biomarkers are involved in ERR (external root resorption). This table presents their names, pathways, and site of detection.

Biomarker	Pathway/Function	Detection Site	References
RANKL/OPG	RANKL/RANK/OPG Signaling Pathway	GCF	Alyahya et al. [37], Fukushima et al. [38], Low et al. [39], Tyrovola et al. [40], Yamaguchi et al. [13]
Interleukin-1B	Inflammatory Pathway	GCF	Bletsa et al. [41], Kulkarni et al. [42], Yao et al. [43]
TNF-Alpha	Inflammatory Pathway	GCF	Kapoor et al. [12], Bletsa et al. [41]
Dentin Sialoprotein (DSP)	BMP/Smad, JNK, ERK, MAPK, and NF-κB signalling	GCF	Ritchie et al. [36], Kumar et al. [35], Kereshanan et al. [44], Lombardo et al. [45]
Dentin phosphoprotein (DPP)	AKT and mTOR	GCF	Mah et al. [34], Silva et al. [46], Yuan et al. [33]
Interleukin-6	Notch Signaling	GCF/PDL	Yamaguchi et al. [13]
MicroRNA-29	Osteoclast regulation	GCF	Atsawasuwan et al. [47]

## References

[1] Bakland L.K. (1992). Root resorption. Dent. Clin. N. Am..

[2] Consolaro A. (2013). The four mechanisms of dental resorption initiation. Dent. Press J. Orthod..

[3] Mavridou A.M., Pyka G., Kerckhofs G., Wevers M., Bergmans L., Gunst V., Huybrechts B., Schepers E., Hauben E., Lambrechts P. (2016). A novel multimodular methodology to investigate external cervical tooth resorption. Int. Endod. J..

[4] Espona J., Roig E., Durán-Sindreu F., Abella F., Machado M., Roig M. (2018). Invasive Cervical Resorption: Clinical Management in the Anterior Zone. J. Endod..

[5] Patel S., Foschi F., Mannocci F., Patel K. (2018). External cervical resorption: A three-dimensional classification. Int. Endod. J..

[6] Patel S., Kanagasingam S., Pitt Ford T. (2009). External cervical resorption: A review. J. Endod..

[7] Aronson J.K., Ferner R.E. (2017). Biomarkers-A General Review. Curr. Protoc. Pharmacol..

[8] Bonnet A., Lagarrigue S., Liaubet L., Robert-Granié C., Sancristobal M., Tosser-Klopp G. (2009). Pathway results from the chicken data set using GOTM, Pathway Studio and Ingenuity softwares. BMC Proc..

[9] Yuryev A., Kotelnikova E., Daraselia N. (2009). Ariadne’s ChemEffect and Pathway Studio knowledge base. Expert. Opin. Drug Discov..

[10] Weltman B., Vig K.W., Fields H.W., Shanker S., Kaizar E.E. (2010). Root resorption associated with orthodontic tooth movement: A systematic review. Am. J. Orthod. Dentofac. Orthop..

[11] Pizzo G., Licata M.E., Guiglia R., Giuliana G. (2007). Root resorption and orthodontic treatment. Review of the literature. Minerva Stomatol..

[12] Kapoor P., Kharbanda O.P., Monga N., Miglani R., Kapila S. (2014). Effect of orthodontic forces on cytokine and receptor levels in gingival crevicular fluid: A systematic review. Prog. Orthod..

[13] Yamaguchi M., Aihara N., Kojima T., Kasai K. (2006). RANKL increase in compressed periodontal ligament cells from root resorption. J. Dent. Res..

[14] George D.I., Miller R.L. (1986). Idiopathic resorption of teeth. A report of three cases. Am. J. Orthod..

[15] Bergmans L., Van Cleynenbreugel J., Verbeken E., Wevers M., Van Meerbeek B., Lambrechts P. (2002). Cervical external root resorption in vital teeth. J. Clin. Periodontol..

[16] Neely A.L., Thumbigere-Math V., Somerman M.J., Foster B.L. (2016). A Familial Pattern of Multiple Idiopathic Cervical Root Resorption With a 30-Year Follow-Up. J. Periodontol..

[17] Neely A.L., Gordon S.C. (2007). A familial pattern of multiple idiopathic cervical root resorption in a father and son: A 22-year follow-up. J. Periodontol..

[18] Macdonald-Jankowski D. (2005). Multiple idiopathic cervical root resorption most frequently seen in younger females. Evid. Based Dent..

[19] Najeeb S., Siddiqui F., Khurshid Z., Zohaib S., Zafar M.S., Ansari S.A. (2017). Effect of bisphosphonates on root resorption after tooth replantation—A systematic review. Dent. Traumatol..

[20] Iwamatsu-Kobayashi Y., Satoh-Kuriwada S., Yamamoto T., Hirata M., Toyoda J., Endo H., Kindaichi K., Komatsu M. (2005). A case of multiple idiopathic external root resorption: A 6-year follow-up study. Oral Surg. Oral Med. Oral Pathol. Oral Radiol..

[21] Llavayol M., Pons M., Ballester M.L., Berástegui E. (2019). Multiple Cervical Root Resorption in a Young Adult Female Previously Treated with Chemotherapy: A Case Report. J. Endod..

[22] Kumar V., Chawla A., Kaur A. (2018). Multiple Idiopathic Cervical Root Resorptions in Patients with Hepatitis B Virus Infection. J. Endod..

[23] Shafi I., Welbury R. (2015). Idiopathic Radiographic Apical Root Resorption in Wind Instrument Players. Dent. Update.

[24] Talebzadeh B., Rahimi S., Abdollahi A.A., Nouroloyuni A., Asghari V. (2015). Varicella Zoster Virus and Internal Root Resorption: A Case Report. J. Endod..

[25] Kjær I., Strøm C., Worsaae N. (2012). Regional aggressive root resorption caused by neuronal virus infection. Case. Rep. Dent..

[26] Von Arx T., Schawalder P., Ackermann M., Bosshardt D.D. (2009). Human and feline invasive cervical resorptions: The missing link?—Presentation of four cases. J. Endod..

[27] Samara E., Kelly E., Walker R., Borumandi F. (2021). Multiple idiopathic cervical root resorption: Case report of an unusual presentation. Spec. Care Dent..

[28] Chen X., Yu X., Yan K., Liu S., Sun Z., Li S. (2020). Multiple idiopathic cervical root resorption involving all permanent teeth. Aust. Endod. J..

[29] Yu V.S., Messer H.H., Tan K.B. (2011). Multiple idiopathic cervical resorption: Case report and discussion of management options. Int. Endod. J..

[30] Warnsinck C.J., Shemesh H. (2018). External cervical root resorption. Ned. Tijdschr. Tandheelkd..

[31] Vieira G.M. (2014). Protein biomarkers of external root resorption: A new protein extraction protocol. Are we going in the right direction?. Dent. Press J. Orthod..

[32] George A., Evans C.A. (2009). Detection of root resorption using dentin and bone markers. Orthod. Craniofac. Res..

[33] Yuan G., Wang Y., Gluhak-Heinrich J., Yang G., Chen L., Li T., Wu L.A., Chen Z., MacDougall M., Chen S. (2009). Tissue-specific expression of dentin sialophosphoprotein (DSPP) and its polymorphisms in mouse tissues. Cell Biol. Int..

[34] Mah J., Prasad N. (2004). Dentine phosphoproteins in gingival crevicular fluid during root resorption. Eur. J. Orthod..

[35] Kumar V., Logani A., Shah N. (2013). Dentine sialoprotein expression in gingival crevicular fluid during trauma-induced root resorption. Int. Endod. J..

[36] Ritchie H. (2018). The functional significance of dentin sialoprotein-phosphophoryn and dentin sialoprotein. Int. J. Oral Sci..

[37] Alyahya L., Myers G.L. (2020). Denosumab Use as a Predictor Variable for External Cervical Resorption: A Case-Control Study. J. Endod..

[38] Fukushima H., Kajiya H., Takada K., Okamoto F., Okabe K. (2003). Expression and role of RANKL in periodontal ligament cells during physiological root-resorption in human deciduous teeth. Eur. J. Oral Sci..

[39] Low E., Zoellner H., Kharbanda O.P., Darendeliler M.A. (2005). Expression of mRNA for osteoprotegerin and receptor activator of nuclear factor kappa beta ligand (RANKL) during root resorption induced by the application of heavy orthodontic forces on rat molars. Am. J. Orthod. Dentofacial. Orthop..

[40] Tyrovola J.B., Spyropoulos M.N., Makou M., Perrea D. (2008). Root resorption and the OPG/RANKL/RANK system: A mini review. J. Oral Sci..

[41] Bletsa A., Berggreen E., Brudvik P. (2006). Interleukin-1alpha and tumor necrosis factor-alpha expression during the early phases of orthodontic tooth movement in rats. Eur. J. Oral Sci..

[42] Kulkarni R.N., Bakker A.D., Everts V., Klein-Nulend J. (2012). Mechanical loading prevents the stimulating effect of IL-1β on osteocyte-modulated osteoclastogenesis. Biochem. Biophys. Res. Commun..

[43] Yao Z., Xing L., Qin C., Schwarz E.M., Boyce B.F. (2008). Osteoclast precursor interaction with bone matrix induces osteoclast formation directly by an interleukin-1-mediated autocrine mechanism. J. Biol. Chem..

[44] Kereshanan S., Stephenson P., Waddington R. (2008). Identification of dentine sialoprotein in gingival crevicular fluid during physiological root resorption and orthodontic tooth movement. Eur. J. Orthod..

[45] Lombardo L., Carinci F., Martini M., Gemmati D., Nardone M., Siciliani G. (2016). Quantitive evaluation of dentin sialoprotein (DSP) using microbeads—A potential early marker of root resorption. Oral Implantol..

[46] Silva T.A., Lara V.S., Silva J.S., Oliveira S.H., Butler W.T., Cunha F.Q. (2005). Macrophages and mast cells control the neutrophil migration induced by dentin proteins. J. Dent. Res..

[47] Atsawasuwan P., Lazari P., Chen Y., Zhou X., Viana G., Evans C.A. (2018). Secretory microRNA-29 expression in gingival crevicular fluid during orthodontic tooth movement. PLoS ONE.

[48] Balducci L., Ramachandran A., Hao J., Narayanan K., Evans C., George A. (2007). Biological markers for evaluation of root resorption. Arch. Oral Biol..

[49] Li Y., Jacox L.A., Little S.H., Ko C.C. (2018). Orthodontic tooth movement: The biology and clinical implications. Kaohsiung J. Med. Sci..

[50] Kagiya T., Nakamura S. (2013). Expression profiling of microRNAs in RAW264.7 cells treated with a combination of tumor necrosis factor alpha and RANKL during osteoclast differentiation. J. Periodontal. Res..

[51] Maegdefessel L., Azuma J., Tsao P.S. (2014). MicroRNA-29b regulation of abdominal aortic aneurysm development. Trends Cardiovasc. Med..

[52] Al-Daghreer S., Doschak M., Sloan A.J., Major P.W., Heo G., Scurtescu C., Tsui Y.Y., El-Bialy T. (2012). Long term effect of low intensity pulsed ultrasound on a human tooth slice organ culture. Arch. Oral Biol..

[53] Yuan G., Yang G., Song G., Chen Z., Chen S. (2012). Immunohistochemical localization of the NH(2)-terminal and COOH-terminal fragments of dentin sialoprotein in mouse teeth. Cell Tissue Res..

[54] Suto M., Nemoto E., Kanaya S., Suzuki R., Tsuchiya M., Shimauchi H. (2013). Nanohydroxyapatite increases BMP-2 expression via a p38 MAP kinase dependent pathway in periodontal ligament cells. Arch. Oral Biol..

[55] Angelova A., Takagi Y., Okiji T., Kaneko T., Yamashita Y. (2004). Immunocompetent cells in the pulp of human deciduous teeth. Arch. Oral Biol..

[56] Yagi Y., Suda N., Yamakoshi Y., Baba O., Moriyama K. (2009). In vivo application of amelogenin suppresses root resorption. J. Dent. Res..

[57] Noda K., Seshima F., Okubo N., Ishii Y., Ota M., Yamada S., Saito A. (2012). Effect of platelet-derived grwth factor-BB on root resorption after reimplantation of partially denuded tooth in dog. Dent. Traumatol..

[58] Ishii Y., Fujita T., Okubo N., Ota M., Yamada S., Saito A. (2013). Effect of basic fibroblast growth factor (FGF-2) in combination with beta tricalcium phosphate on root coverage in dog. Acta. Odontol. Scand..

[59] Shiratani S., Ota M., Fujita T., Seshima F., Yamada S., Saito A. (2012). Effect of basic fibroblast growth factor on root resorption after delayed autotransplantation of tooth in dogs. Oral Surg. Oral Med. Oral Pathol. Oral Radiol..

[60] Hargreaves K., Cohen S., Berman L. (2011). Cohen’s Pathways of the Pulp.

[61] Sharma S., Kumar P., Jain V., Logani A. (2019). Multiple idiopathic cervical root resorption: Diagnosis, clinical/radiographical/histological presentation, and rehabilitation—A 7-year follow-up case report. J. Conserv. Dent..

[62] Wu J., Lin L.Y., Yang J., Chen X.F., Ge J.Y., Wu J.R., Sun W.B. (2016). Multiple idiopathic cervical root resorption: A case report. Int. Endod. J..

